# The intake assessment of diverse dietary patterns on childhood hypertension: alleviating the blood pressure and lipidemic factors with low-sodium seafood rich in omega-3 fatty acids

**DOI:** 10.1186/s12944-020-01245-3

**Published:** 2020-04-07

**Authors:** Anahita Izadi, Leila Khedmat, Reza Tavakolizadeh, Sayed Yousef Motahedi

**Affiliations:** 1 Department of Pediatric Infection Disease, Tehran University of Medical Sciences, Tehran, Iran; 2 Health Management Research Center, Baqiyatallah University of Medical Sciences, Tehran, Iran; 3 Department of Pediatrics, Tehran University of Medical Sciences, Tehran, Iran; 4 Department of Pediatric Nephrology, Bahrami Children Hospital, Tehran University of Medical Sciences, Tehran, Iran

**Keywords:** Children, Pediatric hypertension, Diet, Nutrients, Omega-3 fatty acids, Vitamin D

## Abstract

**Background:**

Childhood hypertension (CH) is related to the dietary intake and diversity of children. The study aimed to assess the critical role of dietary diversity, and seafood long-chain n-3 polyunsaturated fatty acids (LC n-3 PUFAs) in reducing CH among the Iranian community.

**Methods:**

A cross-sectional two-phase study with 7–12-year-old Iranian students was designed. In the initial phase, the socio-demographic characteristics, and blood pressure status (normal, pre-hypertension, and hypertension) based on systolic (SBP) and diastolic (DBP) blood pressure data were assessed. The 24-h dietary recall questionnaire was used to generate the dietary diversity score (DDS, count of consumed food groups) and dietary variety score (DVS, the cumulative number of daily consumed food items). In the second phase, the association between CH reduction and changes in serum 25-hydroxyvitamin D (25OHD), total cholesterol (TC), high-density lipoprotein (HDL), low-density lipoprotein (LDL), and triglycerides (TG) levels of schoolchildren intervened by a seafood diet rich in omega-3 fatty acids were assessed using the regression analyses.

**Results:**

The pre-hypertension and hypertension prevalence rates were 7.8 and 9.15%, respectively. CH was significantly associated with age, gender, and DDS. A significant inverse association was found between the high intake of seafood and CH (*P* = 0.032). The gas-chromatography analysis showed the high presence of α-linolenic (ALA, 6.72%), eicosapentaenoic (EPA, 7.62%), docosapentaenoic (DPA, 5.88%), and docosahexaenoic (DHA, 18.52%) acids in the seafood-based diet (*p* <  0.05). The low blood pressure levels with regular consumption of this healthy-functional diet were significantly associated with a reduction in BMI, LDL, TC, and TG, and a remarkable increase in 25OHD and HDL levels. The multiple linear regression showed that the SBP was highly associated with the TC (*p* <  0.001; β = 0.464).

**Conclusions:**

The age and DDS were efficient predictors for the different CH status. A regular seafood-rich dietary pattern due to the high LC n-3 PUFAs contents could significantly reduce the obesity-related cardiovascular risk factors.

## Introduction

Childhood hypertension (CH) is a growing health problem with a prevalence of pre-hypertension (Pre-HTN, ~ 3.4%) and hypertension (HTN, ~ 3.6%) among pediatrics aged 3 and 18 years [1]. This disorder is much higher in obese children as the Pre-HTN prevalence in obese boys and girls was reported to be 10 and 10.7%, respectively [2]. However, the CH prevalence in different countries and even in various regions of each country may be dissimilar due to the diverse genetics and environmental factors [3, 4]. The most important environmental factors affecting the CH prevalence mainly are socioeconomic status and lifestyle behaviors (e.g., dietary pattern and physical activity) [5]. Low socioeconomic status and air pollution are usually related to distress, mental health problems (such as stress, anxiety, and depression), and health-impairing behaviors [5, 6]. It was demonstrated that there was a high association between childhood hypertension and cognitive dysfunction [7]. Also, the dietary intake of foods containing high fat and sugar, as well as the low level of habitual physical activity in children can potentially increase the risk of developing hypertension during childhood [8]. The blood pressure status is determined through a balance between cardiac output (CO) and vascular resistance (VR). Therefore, an increase in each of these factors (CO or VR) could lead to an enhancement in blood pressure level as a result of changing the homeostasis of body electrolytes such as sodium, calcium, and potassium [9]. CH can occur primarily or secondary. Generally, children’s age and high blood pressure (HBP) were likely to suggest a secondary disorder. The underlying diseases should be initially controlled to treat secondary disorders [10]. Studies revealed that HBP in children could highly result in HTN disorder, cardiovascular diseases (CVDs), stroke, and even early mortality in adulthood [11]. The available evidence also proved that the structural damages to organs, such as thickening the left ventricular wall and pathological changes in vascular walls, are intensified among children with HBP levels [12]. The primarily HBP in these children was associated with other risk factors for CVDs, such as diabetes and hyperlipidemia [11, 13]. Therefore, increasing the awareness and understanding of CH risk factors should be considered as a necessity for the prevention, diagnosis, and treatment of critical underlying diseases.

Childhood and adolescence are crucial life stages to establish individual-related and health behaviors. Behavioral patterns of people in this age range were notably affected their nutritional and health status in the future [14]. Different nutritional indices have been recently introduced to assess the nutritional quality of diets and their role in the prevalence/prevention of many diseases among children and adolescents. Dietary diversity score (DDS) is a valuable indicator of the overall nutritional status. It refers to the nutritional adequacy and dietary diversity so that a higher DDS indicates a more protective effect against CVDs [15]. It has been shown that there was a strong correlation between dietary diversity and the arterial wall index, as well as the prevalence of HBP and obesity [15, 16]. The dietary diversity assessment in children to reduce HTN complications should be critically conducted. There are a few available data to support reliable conclusions related to the fruitful role of diet diversity and functional ingredients in reducing CH. Seafood is one of the most important food groups containing a high number of nutrients with remarkable benefits in human health. This food group contains n-3 polyunsaturated long chain fatty acids (e.g., eicosapentaenoic acid (EPA) and docosahexaenoic acid (DHA)), essential minerals (e.g., potassium, iodine, and selenium), as well as fat- (D, A, and E) and water-soluble vitamins (e.g., B_12_) [17, 18]. The weekly intake of seafood products could well reduce the postprandial triglycerides (TGs), waist circumference, and blood pressure levels, while increased the high-density lipoprotein (HDL) levels [19]. The seafood-based diet was able to maintain cognitive and antidiabetic functions by reducing inflammation [20, 21]. A reduction in the risk of ischemic stroke, congestive heart failure, coronary heart disease, and sudden cardiac death was also observed after the intake of seafoods [22–24]. Hence, the current study aimed to evaluate the relationship between dietary diversity and CH and the nutritional role of some bioactive seafood constitutes in the prevention of HTN among Iranian children aged 7–12 years.

## Methods

**Fig. 1:**
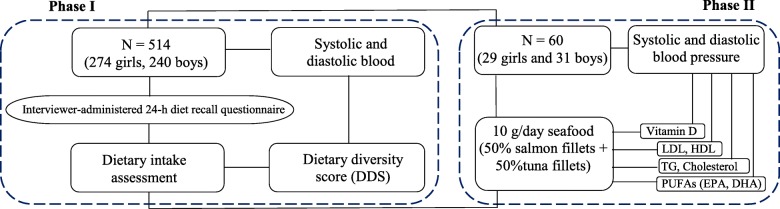
An illustration of two phases conducted in the present study

This study was set up in two research phases (Fig. 1), including (i) the association assessment between dietary diversity and CH, and (ii) the effect evaluation of the main bioactives of the superior nutritional diet in reducing CH.

### Phase I studies

#### Study design and participants

In the first phase, a cross-sectional study between November 2015 and May 2016 with 514 students aged 7–12-years (274 girls and 240 boys) was conducted to investigate the effect of dietary habits on the CH prevalence. Students were chosen among Tehranian children registered in local schools of the 17th district to avoid probable sampling errors. A random number table (RNT) was used to select students after obtaining legal permissions and written informed consent. A two-stage cluster sampling in the first phase was implemented. The first and second sampling stages were the selection of primary schools and elementary students from the first to the sixth grade, respectively.

#### Inclusion and exclusion criteria

Inclusion criteria in the current study were schoolchildren aged 7–12 years old with Iranian nationality. Also, students with specific or chronic diseases such as diabetes, CVDs, kidney and liver diseases, cancer types, food allergies, thyroid problems, epilepsy, and seizure disorders, regular use of any medication or supplement, and unwillingness to continue cooperation with the research program were excluded. Moreover, there was no parental consanguinity in all cases.

#### Socio-demographic and anthropometric data

A face-to-face interview method was used to collect the data of socio-demographic characteristics of students, including age, gender, body mass index (BMI), family’s economic status, and parents’ education level. The height was measured without shoes in a standing position by a stadiometer to the nearest 0.1 cm, whereas the shoulders were in a normal state. A standard, calibrated, electronic balance scale (Seca model 812, Vogel & Halke, Hamburg, Germany) with an accuracy of 0.01 g was used to assess the body weight of children. The BMI was calculated by measuring the weight (in Kg) divided by the square of assessed height (in m).

#### Blood pressure measurement

Systolic (SBP) and diastolic (DBP) blood pressures in the sitting position after a 5 min-rest were determined using a standard mercury pressure gauge with dimensions suitable for children and standard medical devices. In the end, the blood pressure level was divided into three groups: HTN (SBP and/or DBP ≥ 95th percentile + 12 mmHg, or ≥ 140/90 mmHg), elevated or Pre-HTN (SBP and/or DBP ≥ 90th percentile but <95th percentile, or 120/80 mmHg to <95th percentile), and normal (SBP and DBP < 90th percentile or < 120/80 mmHg) [25, 26].

#### Dietary intake assessment

Four trained fieldworkers during lunchtime in schools observed enrolled students and recorded the selected foods, consumed food portions, spilled or exchanged foods and leftovers on dishes in standard forms. They were entirely familiar with the type and preparation method of foods served in each school, including ten various lunch menus along with seasonal fruits and vegetables and also four items of drinks and beverages. An interviewer-administered 24-h diet recall questionnaire was also completed through face-to-face interviews in a randomized order. Two dietitians as interviewers participated in this study. The questionnaire included a list of questions related to food details and quantities (e.g., consumed portion size, ingredients of used foods, and their cooking methods) in the past 24 h [27]. An adult family member (especially the student’s mother) often helped to fill out the questionnaire for the subjects.

#### Determination of dietary diversity score

All the subjects completed a standard dietary diversity questionnaire according to the Food and Agriculture Organization (FAO) guidelines [28]. The diet quality was assessed using the FAO order, based on dietary variety score (DVS) to the cumulative number of various foods used in a day. The standard FAO included nine main food groups: (a) starchy materials and cereals, (b) vitamin A-rich fruits and vegetables, (c) dark green leafy vegetables, (d) other vegetables and fruits, (e) grains, nuts and legumes, dairy products, (f) organ meat (heart, liver, kidney, gizzard, and lung), (g) red meat, fish flesh, and seafoods (h) eggs, and (i) milk and dairy products. The DDS was estimated according to the minimum consumption of at least half serving of one food stated in each group. The DDS also was the total sum of the score of all food groups. Since a score of 1.0 was rated score for each group of consumed foods, the maximum score was 9.0. In this study, the 24-h recall method was used to categorize food groups using the Kant method [29]. In this methodology, the number of food groups out of five main groups with 23 subgroups as DDS was investigated as follows (Table 1):

i. Bread and grains group: white bread, refined grains, cereals, pasta, bread, wheat flour, rice, and flour
ii. Vegetables group: salad vegetables, potatoes, tomatoes, and other edible vegetables
iii. Fruits group: berries, citrus fruits, other fruits, and fruit juices
iv. Meat group: fresh red and poultry meats, fish, shrimp, and eggs
v. Dairy group: milk, yogurt, doogh, and cheese

**Table 1:** A summary of 24-h dietary recall questionnaire data in the studied schoolchildren in normal, Pre-HTN, and HTN groups

Food group(s)	Food items (preparation method)	Normal (***n*** = 429)			Pre-HTN (***n*** = 47)			HTN (***n*** = 40)		
		R_consuming_ (n)^a^	MDI (g)^b^	Energy (cal)	R_consuming_ (n)^a^	MDI (g)^b^	Energy (cal)	R_consuming_ (n)^a^	MDI (g)^b^	Energy (cal)
Bread and grains	refined cereals (e.g., cake, white bread, pasta, biscuits, refined grain breakfast cereals, white rice, and pancakes), and whole cereal grains (e.g., wheat, corn, and rice)	429	325 ± 28	630 ± 72	47	318 ± 32	603 ± 82	40	320 ± 41	612 ± 35
Vegetables	salad vegetables (e.g., broccoli, cabbage, cauliflower, mushrooms, bell peppers, carrots, celery, cucumbers, lettuce, peas, spinach and tomatoes), and cooked/fried potatoes	297	214 ± 12	138 ± 27	32	224 ± 22	145 ± 41	28	215 ± 17	140 ± 29
Fruits	berries (e.g., strawberries, raspberries, blueberries, blackberries, and white and red currants), banana, citrus fruits (e.g., orange, tangrine, kiwi, and limon), apple, and fruit juices	298	337 ± 15	196 ± 52	32	341 ± 21	241 ± 27	25	334 ± 18	232 ± 10
Milk and dairy products	cow milk, cream, butter, Feta cheese, yogurt, Doogh, and Kashk	110	422 ± 75	643 ± 36	8	412 ± 74	654 ± 45	10	420 ± 45	635 ± 33
Meat and egg products	red meat (cooked/grilled), poultry meat (cooked/fried), and egg (boiled/pan-fried)	327	167 ± 11	240 ± 15	37	172 ± 13	254 ± 26	32	185 ± 9	267 ± 20
Seafoods	fried/grilled fish (e.g., salmon, trout and tuna, and carp), and fried shrimp	112	376 ± 28	472 ± 51	20	291 ± 19	318 ± 37	8	252 ± 32	248 ± 28
Junk foods	salted snacks, fried fast foods, sugary carbonated beverages, gum, candy, and sweet desserts	164	175 ± 8	342 ± 14	18	180 ± 6	351 ± 14	13	154 ± 11	302 ± 16

^a^ R_consuming_ (n): Respondents with consumption (number), ^b^MDI: Mean daily intake (gram per person consuming these foods)

### Phase II studies

#### Study design and subjects

In the second phase, a cross-sectional design between April 2016 and June 2016 was carried out with 60 students (29 girls and 31 boys) with a regular daily intake of seafood. The ages of total students, girls, and boys in this study phase were 8.65 ± 0.12, 8.69 ± 0.45, and 8.62 ± 0.32 years, respectively. Parents were asked to ensure that their children averagely consumed 10 g of low-sodium seafood per day with fish fillets of Atlantic salmon (*Salmo salar*) (50%) and tuna (50%) for 60 days. These fish fillets in the first phase were frequently consumed by students with having a seafood-based diet pattern, probably due to their high accessibility in the market. Overall, fish fillets were processed with different salt levels to increase their shelf life during the cold storage. Accordingly, the unwrapped fresh fish fillets without any added salt as an agent increasing the blood pressure were bought from the aquaculture centers in Tehran. The subjects were selected with a non-random and available sampling method from the primary population to assess the effect of the main bioactive nutrients present in this food group on the CH.

#### Chemicals and reagents

All the used chemicals in the analysis of fatty acids were purchased from Merck Chemical Co. (Darmstadt, Germany).

#### Fatty acids analysis of seafood-based diet

The method of Joseph and Ackman [30] with slight modifications was used to analyze fatty acids of recommended low-sodium seafood-based diet. In brief, the seafood oil was extracted in a Soxhlet apparatus using the extractant of petroleum ether. About 25.0 mg of oil in a 20 mL capped centrifuge tube was mixed with 1.5 mL of 0.5 M NaOH in methanol, heated in a water bath (100 °C, 5 min), and cooled at 22 ± 1 °C. Two milliliters of 12% boron trifluoride in methanol was added to the prepared mixture, re-heated (100 °C, 30 min), and then cooled before adding 1.0 mL of isooctane. In the next step, the solution was strongly stirred for 1.0 min before mixing with the saturated solution of NaCl (5.0 mL) to increase the phase separation rate. The esterified mixture was placed in the refrigerator to separate two phases, and a syringe then removed the supernatant. A mixture containing 10.0 mL of isooctane and 0.05% BHT (as an antioxidant agent) was transferred to the remaining phase into the tube, stirred to collect the formed supernatant. The sample concentrated up to 1.0 mL was then injected into the SP-2560 capillary column (100 m length × 250 μm internal diameter, 0.2 μm of film) of gas chromatography coupled with flame ionization detector (FID) (Varian 3400; Varian, Palo Alto, CA, USA). The temperature program of the column ramp was set up in the following steps: (i) the initial temperature was 70 °C, (ii) heated to 140 °C (20 °C/min) and held for 1 min; (iii) then to 180 °C (4 °C/min) and held for 1 min; and (iv) then to 225 °C (3 °C/min) and held for 30 min. The gasifying temperature was 250 °C. Nitrogen was used as the carrier gas at a flow rate of 1.0 mL/min. The injection volume was 1.0 μL in a split mode of injection at a ratio of 45:1. A comparison between the retention time of the fatty acid methyl esters (FAMEs) and their authentic standard mixtures was used to identify fatty acids. Also, the area under the peak data was used to calculate the contribution of each fatty acid as a percentage of total fatty acids using the Varian Star Chromatography Software (Varian Star WS 5.31; Varian Inc., CA, USA) [31].

#### Assessment of serum 25-hydroxyvitamin D

After a 12 h fasting period, antecubital venous blood samples were collected from all of the selected schoolchildren at 7:00–8:00 a.m. Serum levels of 25-hydroxyvitamin D (25OHD, ng/mL) were measured according to the procedure described by Fasihpour et al. [32] with minor modifications. In brief, the sera separation was performed by centrifugation at 3500 rpm for 25 min and then transferred into labeled polypropylene tubes to be stored at − 20 °C until the analysis time of 25OHD using a commercially available enzyme-linked immunosorbent assay (ELISA) kit (DiaSource, Belgium).

#### Measurement of lipidemic factors

The antecubital-venous blood samples of all the students were taken after an overnight fast (minimum 12 h). The total cholesterol (TC), HDL, low-density lipoprotein (LDL), and TG were measured by enzymatic methods using an Elan 2000 autoanalyzer (Eppendorf Co., Frankfurt, Germany) and expressed as mg/dL.

#### Statistical analysis

The obtained data were analyzed using SPSS 22.0 software (SPSS, Inc., Chicago, IL, USA). The analysis of variance (ANOVA) was employed to assess the significant quantitative differences among the studied data. The one-sample Kolmogorov-Smirnov test was used to evaluate the normality of variables’ distribution. Data were examined at a significance level of *p* <  0.05. Pearson’s test was also carried out to find any correlation coefficient between tested variables. Associations between SBP and factors of serum 25OHD, DBP, TC, TG, LDL, and HDL were also assessed using the multiple linear regression analysis (LRA) in multivariate adjustment with two possible confounders; i.e., age and BMI.

## Results

### Phase I-related findings

**Fig. 2:**
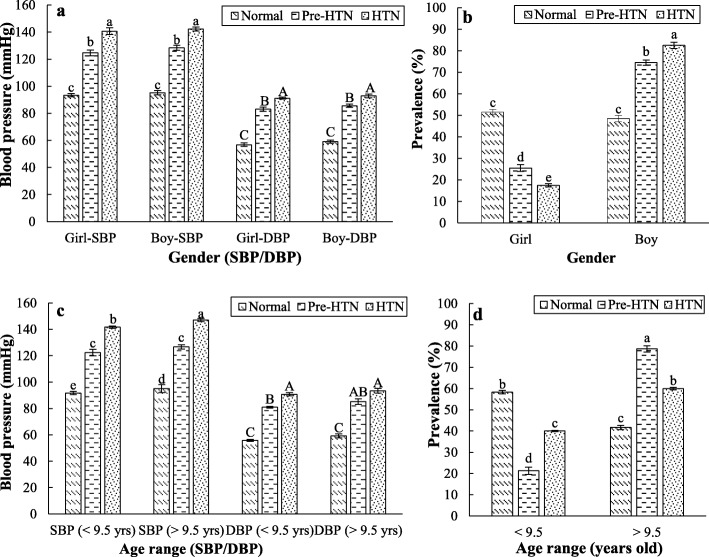
The relationship of blood pressure levels (**a**, **c**) and CH prevalence (**b**, **d**) with gender (**a**, **b**) and age (**c**, **d**) in the considered triple children groups (HTN (SBP and/or DBP ≥ 140/90 mmHg); Pre-HTN (SBP and/or DBP ≥ 120/80 mmHg); Normal (SBP and/or DBP < 120/80 mmHg)). Values with different statitical letters (a-c or A-C) are significant (*p* < 0.05)

**Fig. 3:**
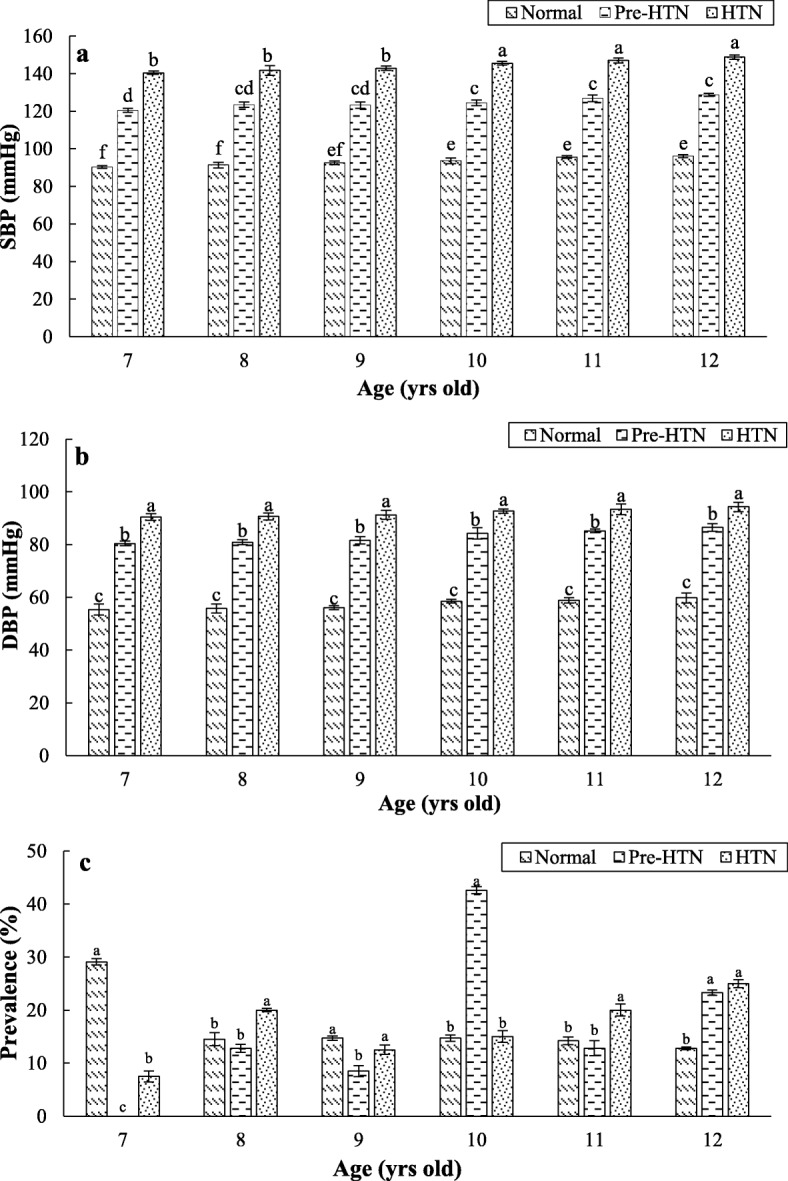
The association of children’s age with their blood pressure levels (**a**, SBP; **b**, DBP) and CH prevalence rate (**c**) in the triple groups (HTN (SBP and/or DBP ≥ 140/90 mmHg); Pre-HTN (SBP and/or DBP ≥ 120/80 mmHg); Normal (SBP and/or DBP < 120/80 mmHg)). Values with different statitical letters (a-c or A-C) are significant (*p* < 0.05)

The prevalence of Pre-HTN and HTN in students aged 7–12 years old was determined to be 9.1 and 7.8%, respectively. The CH also was significantly correlated with the gender (*p* <  0.001), so that the more Pre-HTN and HTN rate in boys compared to girls was observed (Fig. 2a and b). There was an association between students’ age and high risk of HTN disorders (p <  0.001, Fig. 3). The students aged 9.5 or more showed higher rate of Pre-HTN (78.7%) and HTN (60.0%) (*p* = 0.001, Fig. 2c and d). Not only a considerable prevalence rate of Pre-HTN (42.6%) was detected for students aged 10 years old, but also children’s 12-year-old age group showed a high Pre-HTN (23.3%) and HTN (25.0%) rate (Fig. 2c). The lowest and highest rates of HTN were found in the age groups of 7 and 12 years, respectively (Fig. 3). The mean SBP and DBP levels for children aged 7 years in normal, Pre-HTN, and HTN groups were 90.3 and 55.4 mmHg, 120.5 and 80.4 mmHg, and 140.5 and 90.5 mmHg, respectively. The corresponding values for children aged 12 years were 96.1 and 59.8 mmHg, 128.7 and 86.5 mmHg, and 148.8 and 94.3 mmHg, respectively. Therefore, there were significant differences in levels of SBP and DBP among schoolchildren in the different age groups (*p* <  0.05, Fig. 3). Moreover, the minimum (7.5%) and maximum (25.0%) number of hypertensive cases belonged to the children aged 7 and 12 years, respectively (Fig. 3).

**Fig. 4:**
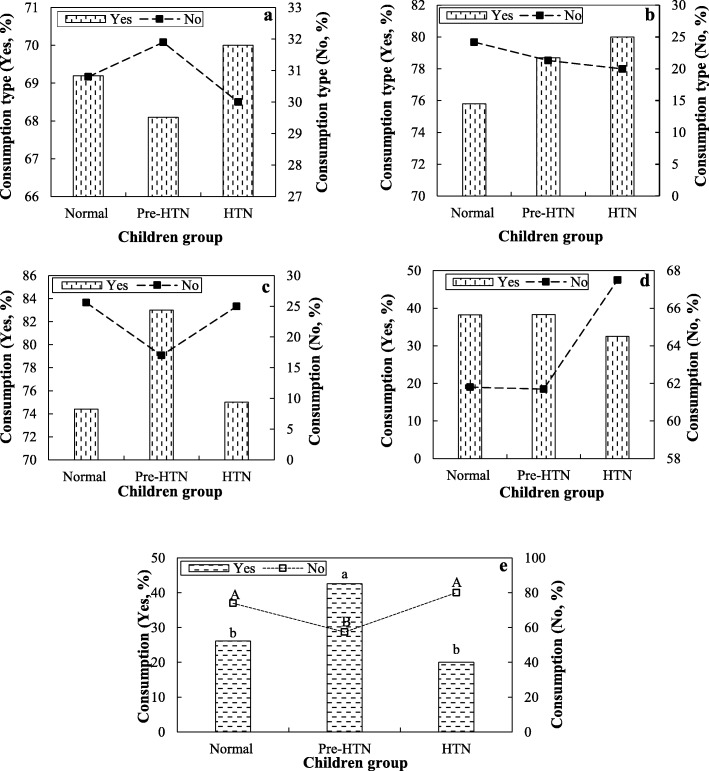
The association between the CH prevalence and the consumption of fruits and vegetables (**a**), red meats (**b**), dairy products (**c**), junk foods (**d**), and seafoods (**e**; values with different statitical letters (a-b/A-B) are significant (*p* < 0.05)) in the different children groups (normal, pre-HTN, and HTN)

Table 1 shows the consumption of food groups, including fruits, vegetables, red meats, dairy products, junk foods, and seafoods in normal, Pre-HTN, and HTN children groups. The amounts of mean daily intake (MDI) and received energy of each food group were also specified (Table 1). All the children in three groups of normal, Pre-HTN, and HTN consumed grains and breads. However, the consumption of other food groups in the triple groups was different. The MDI levels of breads/grains, vegetables, fruits, egg/meat products, dairy products, seafoods, and junk foods in the different groups were 318–325, 214–224, 334–341, 167–185, 412–422, 252–376, and 154–180 g, respectively (Table 1). The total absorbed calories by schoolchildren in normal, Pre-HTN, and HTN groups were 2661, 2566, and 2436 cal, respectively. Moreover, Fig. 4 depicts the association between the CH prevalence and the consumption of food groups, in normal, Pre-HTN, and HTN children groups. There was no significant relationship between the CH reduction and the consumption of red meats (*p* = 0.769), dairy products (*p* = 0.430), fruits and vegetables (*p* = 0.980), as well as junk foods (*p* = 0.772) (Fig. 4a-d). However, the consumption of seafoods among different food groups had a significant association with the prevalence reduction of CH (*p* = 0.032; Fig. 4e).

**Fig. 5:**
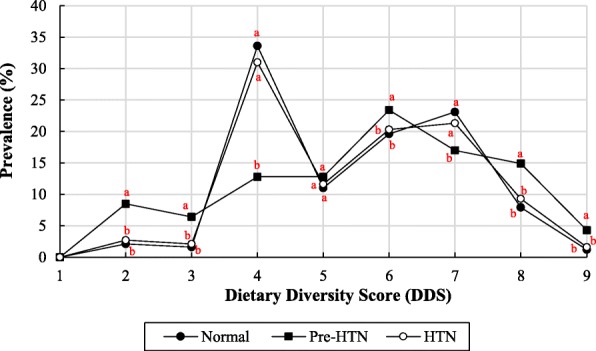
The association between the DDS and CH prevalence among the different children groups (HTN (SBP and/or DBP ≥ 140/90 mmHg); Pre-HTN (SBP and/or DBP ≥ 120/80 mmHg); Normal (SBP and/or DBP < 120/80 mmHg)). Values with different statitical letters (a-c) in the same DDS are significant (*p* < 0.05)

Figure 5 illustrates the association between the DDS and CH prevalence among the different children groups. The DDS value was significantly different in normal, Pre-HTN, and HTN children groups (*p* < 0.004). There was a significant association between the DDS value (especially, ≥ 7.0) and the reduction of CH prevalence (*p* = 0.004). A notable increase in CH prevalence in normal and HTN groups at a DDS of 4.0 was found. Nonetheless, the maximum CH prevalence for children in the Pre-HTN group was identified at a DDS of 6.0 (Fig. 5).

### Phase II related-findings

The lipid content of the seafood diet was 13.72%. The GC analysis revealed that the main fatty acids present in seafood diet were linoleic acid (18:2; 3.65%), α-linolenic acid (ALA, 18:3; 6.72%), EPA (20:5; 7.62%), docosapentaenoic acid (DPA, 22:5; 5.88%), and DHA (22:6; 18.52%). There was a significant difference in the content of fatty acids present in the seafood diet (*p* < 0.05). Table 2 showed that the low-sodium seafood-based dietary treatment could significantly reduce BMI, SBP, DBP, LDL, TG, and TC levels in boys, girls, and the total population (*p* < 0.05). Also, this nutritional intervention increased the HDL and 25OHD levels in all the populations significantly (*p* < 0.05). There was no significant difference in BMI, 25OHD, blood pressure, and lipidemic factors after the intervention among boys, girls, and total populations (Table 2). However, an insignificantly higher 25OHD, SBP, LDL, TG, and TC values in boys compared to girls were observed (Table 2).

**Table 2:** A comparative study on levels of BMI, blood pressure, serum vitamin D, and lipidemic factors among girls (*n* = 29), boys (*n* = 31), and total population (*n* = 60) before and after the low-sodium seafood-based dietary intervention^a^

Parameter^b^	Boys		Girls		Total	
	Before	After	Before	After	Before	After
BMI (kg/m^2^)	17.53 ± 0.32^a^	17.07 ± 0.21^b^	17.25 ± 0.18^a^	17.00 ± 0.22^b^	17.41 ± 0.19^a^	17.04 ± 0.21^b^
25OHD (ng/mL)	17.89 ± 3.54^b^	20.49 ± 5.34^a^	16.77 ± 3.49^b^	18.37 ± 3.84^a^	17.74 ± 3.95^b^	19.46 ± 4.76^a^
SBP (mmHg)	106.71 ± 0.26^a^	102.64 ± 0.68^b^	105.41 ± 0.22^a^	102.02 ± 1.03^b^	106.02 ± 0.31^a^	102.34 ± 0.91^b^
DBP (mmHg)	62.37 ± 0.08^a^	60.92 ± 0.52 ^b^	62.97 ± 0.34^a^	60.93 ± 0.57^b^	62.54 ± 0.27^a^	60.92 ± 0.54^b^
LDL (mg/dL)	109.54 ± 0.85^a^	99.92 ± 4.71^b^	107.42 ± 0.61^a^	98.70 ± 5.72^b^	108.05 ± 0.74^a^	99.33 ± 5.21^b^
HDL (mg/dL)	32.26 ± 1.75^b^	39.83 ± 5.26 ^a^	31.75 ± 1.57^b^	40.20 ± 6.11^a^	32.09 ± 1.28^b^	40.01 ± 5.64^a^
TG (mg/dL)	88.25 ± 4.04^a^	76.09 ± 7.43^b^	83.32 ± 3.77^a^	75.80 ± 9.30^b^	84.65 ± 2.98 ^a^	75.95 ± 8.31^b^
TC (mg/dL)	182.32 ± 2.65^a^	169.83 ± 7.73^b^	184.23 ± 2.24^a^	169.17 ± 9.76^b^	181.29 ± 1.89^a^	169.51 ± 8.70^b^

^a^ The mean age of boys, girls, and total population was 9.02 ± 1.60, 9.31 ± 1.47, and 9.16 ± 1.53 years, respectively

^b^ The means (in each row) for each children’s group with dissimilar letters (a,b) are significantly different (*p* < 0.05)

Table 3 reveals the results of the Pearson’s correlation test between age, BMI, blood pressure (i.e., SBP and DBP), lipid profile (i.e., LDL, DHL, TG, and TC), and vitamin D amounts in the study population. A highly significant correlation was explored among all the investigated parameters. Nonetheless, there was no significant association between serum 25OHD and age. The maximum correlation was detected between LDL and HDL (*p* < 0.001; *r* = − 0.936). Conversely, the lowest significant correlation rate was identified between LDL/HDL and age, as well as 25OHD and SBP. In general, a negative, significant correlation was found between 25OHD/HDL levels and other parameters (i.e., age, BMI, DBP, SBP, LDL, TG, and TC). Table 4 also exhibits the multiple linear regression results of all the variables as a function of SBP. According to the fitted model (R^2^ = 0.825; adjusted R^2^ = 0.794), the effect of gender, DBP, HDL, and TC on the SBP changes was highly significant. Among these variables, TC had the highest role in SBP changes. For every 1 mg/dL increase in TC, the SBP was expected to rise by 0.464 mmHg. However, the LDL, BMI, TG, age, and 25OHD were not significantly associated with the SBP.

**Table 3:** Correlation study between age, BMI, blood pressure, lipid profile and vitamin D levels in the study population

Pearson’ test	Age (yrs. old)	BMI (kg/m^**2**^)	SBP (mmHg)	DBP (mmHg)	LDL (mg/dL)	HDL (mg/dL)	TG (mg/dL)	TC (mg/dL)	25OHD (ng/mL)
**Age (yrs. old)**	1	*r* = 0.553	*r* = 0.452	*r* = 0.538	*r* = 0.303	*r* = − 0.283	*r* = 0.719	*r* = 0.406	*r* = − 0.252
		*p* < 0.001	*p* < 0.001	*p* < 0.001	*p* = 0.019	p = 0.028	*p* < 0.001	p = 0.001	*p* = 0.052^ns^
**BMI (kg/m**^**2**^**)**	*r* = 0.553	1	*r* = 0.757	*r* = 0.728	*r* = 0.530	*r* = − 0.545	*r* = 0.599	*r* = 0.739	*r* = − 0.340
	*p* < 0.001		*p* < 0.001	*p* < 0.001	*p* < 0.001	*p* < 0.001	*p* < 0.001	*p* < 0.001	*p* = 0.008
**SBP (mmHg)**	*r* = 0.452	*r* = 0.457	1	*r* = 0.723	*r* = 0.642	*r* = − 0.643	*r* = 0.643	*r* = 0.793	*r* = − 0.295
	*p* < 0.001	*p* < 0.001		*p* < 0.001	*p* < 0.001	*p* < 0.001	*p* < 0.001	*p* < 0.001	p = 0.022
**DBP (mmHg)**	*r* = 0.528	*r* = 0.728	*r* = 0.723	1	*r* = 0.559	*r* = − 0.574	*r* = 0.621	*r* = 0.712	*r* = − 0.371
	*p* < 0.001	*p* < 0.001	*p* < 0.001		*p* < 0.001	*p* < 0.001	*p* < 0.001	*p* < 0.001	*p* = 0.004
**LDL (mg/dL)**	*r* = 0.303	*r* = 0.530	*r* = 0.642	*r* = 0.559	1	*r* = − 0.936	*r* = 0.772	*r* = 0.564	*r* = − 0.658
	p = 0.019	*p* < 0.001	*p* < 0.001	*p* < 0.001		*p* < 0.001	*p* < 0.001	*p* < 0.001	*p* < 0.001
**HDL (mg/dL)**	*r* = − 0.283	*r* = − 0.545	*r* = − 0.643	*r* = − 0.574	*r* = − 0.936	1	*r* = − 0.720	*r* = − 0.581	*r* = 0.673
	*p* = 0.028	*p* < 0.001	*p* < 0.001	*p* < 0.001	*p* < 0.001		*p* < 0.001	*p* < 0.001	*p* < 0.001
**TG (mg/dL)**	*r* = 0.719	*r* = 0.599	*r* = 0.643	*r* = 0.621	*r* = 0.772	*r* = − 0.720	1	*r* = 0.573	*r* = − 0.526
	*p* < 0.001	*p* < 0.001	*p* < 0.001	*p* < 0.001	*p* < 0.001	*p* < 0.001		*p* < 0.001	*p* < 0.001
**TC (mg/dL)**	*r* = 0.406	*r* = 0.739	*r* = 0.793	*r* = 0.712	*r* = 0.564	*r* = − 0.581	*r* = 0.573	1	*r* = − 0.329
	p = 0.001	*p* < 0.001	*p* < 0.001	*p* < 0.001	*p* < 0.001	*p* < 0.001	*p* < 0.001		*p* = 0.010
**25OHD (ng/mL)**	*r* = − 0.252	*r* = − 0.340	*r* = − 0.295	*r* = − 0.371	*r* = − 0.658	*r* = 0.673	*r* = − 0.526	*r* = − 0.329	1
	p = 0.052^ns^	p = 0.008	*p* = 0.022	p = 0.004	*p* < 0.001	*p* < 0.001	*p* < 0.001	p = 0.010	

*ns* non-significant

**Table 4:** The multiple linear regression analysis of determinants of systolic blood pressure (SBP)^a^

Model	Unstandardized coefficients		Standardized β	*t*	p-value ^b^
	β	SE			
Constant	67.363	8.434	–	7.987	< 0.001
Age (year)	0.074	0.042	0.123	1.778	0.081^ns^
BMI (kg/m^2^)	0.446	0.438	0.106	1.019	0.313^ns^
Gender	−0.575	0.108	−0.316	−5.322	< 0.001
DBP (mmHg)	0.474	0.148	0.280	3.19	0.002
HDL (mg/dL)	−0.033	0.012	−0.203	−2.677	0.010
TG (mg/dL)	0.004	0.015	0.039	0.288	0.775^ns^
TC (mg/dL)	0.049	0.009	0.464	5.248	< 0.001
25OHD (ng/mL)	0.015	0.016	0.077	0.914	0.365^ns^
Excluded variable	β	t	p-value ^b^	Partial correction	Tolerance
LDL (mg/dL)	−0.059	−0.257	0.775^ns^	−0.041	0.064

^a^ Multiple linear regression model: R^2^ = 82.5%, adjusted R^2^ = 79.4%

^b^*p*-value < 0.05 is significant, ns: non-significant

## Discussion

The prevalence of Pre-HTN and HTN among 12–7-year aged students was reported to be 9.1 and 7.8%, respectively. The similar prevalence rate for CH in other cities of Iran for the same age groups was reported. Mehr-Alizadeh et al. [33] reported an HTN prevalence rate of 3–9.6% for 7–17-year aged students in Semnan city. Also, Zardast et al. [34] estimated a prevalence rate of 11.6 and 7.4% for Pre-HTN and HTN in 6–11-year aged children in Birjand city, respectively. It seems that the economic situation was critical in CH development. Since the 17th district located in the southern part of Tehran had a similar economic situation to most cities of Iran, the results obtained in Semnan and Birjand cities were in line with our findings. The age elevation was associated with an increased HTN risk. Similarly, some researchers found that higher age was related to a rise in the CH prevalence [35, 36]. Patil and Garg [37] and Hakim et al. [38] also mentioned that the children’s age was associated with their average SBP and DBP. An integration between nutritional diet and physical activity pattern in students 7 years old might prevent the prevalence of Pre-HTN. It seems that Iranian parents in the first year of school had serious attention to their children’s diet and exercise. However, reasons for higher pre-HTN prevalence at ages more than 7 years old might be related to lower attention of parents to the accurate lifestyle of students by increasing their age in terms of high-fat diets and lower physical activities. As a result, this increase may be owing to the increase in body mass, which occurs as the child grows up. However, this fact in adult populations was attributed to the function reduction of the body’s antioxidant enzymes (e.g., superoxide dismutase) by aging. The HTN was also increased with a decrease in the synthesis of nitric oxide as the body’s vasodilator [39]. The significantly higher HTN prevalence in boys than girls was found. This was consistent with the study of Brady et al. [8] in the US, who studied the HTN prevalence among 184 children aged 3–20 years. However, the significantly higher prevalence of HTN in girls compared to boys in Shiraz, Iran, was earlier reported by Ayatollahi and Zare [40]. The difference in lifestyle, diet patterns, and physical activities might be involved in the different prevalence of HTN in girls and boys before puberty.

According to the received energy data, the calorie intake levels in the 24-h recall method was more than the recommended calorie levels of the DASH diet. This result may be due to the higher consumption of junk foods and the lower number of seafood-related servings. Based on the dietary recommendations of therapeutic lifestyle changes (TLC) and dietary approaches to stop hypertension (DASH), receiving the food groups of fruits and vegetables (high content of potassium, magnesium, stanols, sterols, and fibers), as well as dairy products (remarkable amounts of calcium and vitamin D) had an important role in reducing CH. In contrast, the meat group had a direct relationship with the CH prevalence due to the high content of sodium and saturated fatty acids (SFAs). However, we found that the consumption of diets based on fruits, vegetables, and dairy products, along with meats, had not any significant impact on the prevalence of HTN and Pre-HTN in children. It was indicated that the imbalance of mineral metabolism (i.e., calcium, potassium, and sodium) in the daily intake of food groups is a more significant factor in the HTN prevalence [41]. This result was probably owing to the low efficiency of the methodology (Kant method) used in the current study to determine the dietary groups. It was recommended to daily receive more than five servings of fruits and vegetables for reducing or controlling the HTN risk. However, only half-servings per day from each child’s diet group in the current research was assigned to this food group. Besides, the increased SFAs, cholesterol, and hidden salt content in high-fat dairy products might notably eliminate their beneficial effects on the status of blood pressure. Similarly, the group of red meats might have destructive effects on the blood pressure because of the high content of sodium and SFAs. It was hypothesized that the CH could be directly escalated by consuming junk foods (e.g., chips, puffs, carbonated beverages, and sodas) as a result of high amounts of calories and sodium. Stanley et al. [42] considered the high content of sugar, fat, and sodium in junk foods as possible risk factors for HBP. Nonetheless, this theory was not demonstrated because we found that the consumption of junk foods has not any significant relationship with the occurrence of HTN and Pre-HTN in children. On the other hand, the consumption effects of seafood on CH reduction were because of the beneficial role of omega-3 fatty acids such as the vascular response to angiotensin, the improvement of vascular function, and the inflammation moderation or the attenuation of proinflammatory reactions involved in hypertension [43–45]. Seafood is also considered as a good protein source with high digestibility, bioavailability, and efficiency ratio. The presence of essential amino acids (e.g., cystine, lysine, methionine, threonine, and tryptophan) in the structure of angiotensin I–converting enzyme inhibitory peptides may reduce the CH [46]. The consumption of seafood products through high daily intake of dietary potassium is associated with the reduced ratio of sodium (Na) to potassium (K) and blood pressure levels in children [47]. However, the synergy effect of different nutrients of seafood sources on the CH reduction has been less investigated.

The higher dietary diversity can cause a lower calorie density of consumed foods. Even though the DDS was significantly different in normal, Pre-HTN, and HTN children, a direct or reverse relationship for this difference was not generalizable. However, most children in both groups of normal and Pre-HTN received a DDS score of 4.0. Results of this study were in line with findings reported by Lazarou et al. [48], who explored the relationship between dietary patterns and blood pressure levels using the E-KINDEX scoring system. In this methodology, the consumed foods were divided into 13 groups and individual points from their receipt. Results indicated that the E-KINDEX score of foods had been independently linked to lower blood pressure among healthy children. Also, our findings were consistent with the results of Miller et al. [49] as there was not a link between dietary diversity and the prevalence of hypertension. They explained that the imbalance exists in the dietary intake which could significantly lead to the prevalence of hypertension [49]. Obesity is one of the most critical risk factors for hypertension. It is strongly advised to reduce the weight and to receive less high-quality foods in the nutritional medicine for preventing and treating hypertension in children. The higher diet diversity could cause a lower calorie density of consumed food [50]. However, despite the significant difference in DDS value on the status of blood pressure in children, there was no linear relationship among normal, pre-hypertension and hypertension children. Thus, higher or lower DDS did not necessarily mean an association with the hypertension status of children. As a result, it was suggested that not only the scoring method used by the FAO should be implemented, but also the dietary quality score (DQS) should be calculated.

The intake of a low-sodium seafood-based diet could significantly reduce the levels of BMI, blood pressure, and adverse lipidemic factors (such as LDL, TG, and TC) in the study population. In contrast, the serum vitamin D and HDL levels were remarkably increased after implementing the seafood-based dietary intervention. Tu et al. [51] mentioned that obesity and body fat in children were directly associated with their HBP. Therefore, fish oil and omega-3 supplementation can be used as complementary therapies for BMI reduction in obese and overweight patients with HTN and CVDs. Earlier, Kodas et al. [52] in animal studies pointed out that the presence of long-chain n-3 polyunsaturated fatty acids (LC n-3 PUFAs) could modulate the function of the major brain neurotransmitters such as noradrenaline secretion, as well as serotonin and dopamine systems, which are usually involved in the adjustment of blood pressure and heart rhythm. Baltzell et al. [53] explained that the enzyme of lipoprotein lipase (LPL) located in the endothelial layer of capillaries in the muscle and adipose tissues had a high potential to hydrolyze chylomicron- and very-LDL (VLDL)-triacylglycerol to their structural fatty acids. Although the intake of LC n-3 PUFAs with the fish consumption elevated the activity of muscle LPL, the adipocyte LPL activity was decreased. Thus, the changed LPL activities were along with a reduction in body fat and plasma TG levels, showing the TG utilization was altered from storage form in adipocytes to oxidized form in muscles after the consumption of LC n-3 PUFAs [54]. In addition, the hypocholesterolemic effects of fish proteins accompanied by lower TG levels were previously reported [55, 56].

### Limitations

There were some limitations to the present study. It seems that Kant’s method was inadequate to assess the influence of food groups on the levels of blood pressure in children. For example, it is suggested that the type of consumed meat will be considered in two marine and non-marine groups in future studies. The use of weekly intake as a criterion for the intake level assessment of junk foods likely was not sufficiently useful. Besides, the consumption recording of such foods in the interview possibly resulted in an increase in the response error due to the difficulty of remembering the time of day of food intake. It is recommended to use a feed registration method or a 24-h recall questionnaire in 3 days (two days a week and one weekend) to evaluate the weekly intake for junk foods. Also, there was a strict requirement for further high-quality population-based investigations and randomized controlled trials of long duration to comprehensively assess the effects of LC n-3 PUFAs and fish on renal function and its role in reducing the HTN rate.

## Conclusion

This study showed that high dietary diversity could be a powerful tool in reducing the HTN prevalence rate among Iranian schoolchildren. The intake of junk foods, fruits and vegetables, red meats, and dairy products were not significantly correlated with CH. However, a significant association between seafood intake and reduced blood pressure demonstrated the nutritional efficiency of LC n-3 PUFAs in reducing the children’s blood pressure levels. The regular consumption of a healthy seafood-based diet could significantly reduce HTN and BMI due to the high presence of ALA, EPA, DPA, and DHA, the increased 25OHD and HDL levels, and the reduced amounts of LDL, TC, and TG. The future practical nutritional programs should be settled on the effectiveness of different seafood-based dietary patterns in the prevention of CH, overweight, and obesity as risk factors for CVDs. The choice of innovative non-thermal cooking ways for the preservation of nutritional constitutes present in seafood products is necessary as it has an influential role in childhood nutrition and wellbeing. Severe attention should be paid towards the implementation of case-control studies with an extensive follow up period to evaluate long-term effects of different seafood products and their nutritional benefits. Finally, more attention in the public health framework should be directed to improve dietary diversity and preparation methods of particular food items.

## Data Availability

The datasets used and/or analyzed during the current study are available from the corresponding author on reasonable request.

## Abbreviations

CH: Childhood hypertension
LC n-3 PUFAs: Long-chain n-3 polyunsaturated fatty acids
DDS: Dietary diversity score
DVS: Dietary variety score
DQS: Dietary quality score
MDI: Mean daily intake
25OHD: 25-hydroxyvitamin D
TC: Total cholesterol
HDL: High-density lipoprotein
LDL: Low-density lipoprotein
VLDL: Very-low-density lipoprotein
LPL: Lipoprotein lipase
TG: Triglyceride
BMI: Body mass index
ALA: α-linolenic acid
EPA: Eicosapentaenoic acid
FAMEs: Fatty acid methyl esters
DPA: Docosapentaenoic acid
DHA: Docosahexaenoic acid
Pre-HTN: Pre-hypertension
HTN: Hypertension
CO: Cardiac output
VR: Vascular resistance
HBP: High blood pressure
CVDs: Cardiovascular diseases
RNT: Random number table
SBP: Systolic blood pressure
DBP: Diastolic blood pressure
ELISA: enzyme-linked immunosorbent assay
TLC: Therapeutic lifestyle changes
DASH: Dietary approaches to stop hypertension
SFAs: Saturated fatty acids
