# Correction to: The intake assessment of diverse dietary patterns on childhood hypertension: alleviating the blood pressure and lipidemic factors with low-sodium seafood rich in omega-3 fatty acids

**DOI:** 10.1186/s12944-020-01396-3

**Published:** 2020-10-21

**Authors:** Anahita Izadi, Leila Khedmat, Reza Tavakolizadeh, Sayed Yousef Mojtahedi

**Affiliations:** 1 Department of Pediatric Infection Disease, Tehran University of MedicalSciences, Tehran, Iran; 2 Health Management Research Center, BaqiyatallahUniversity of Medical Sciences, Tehran, Iran; 3 Department of Pediatrics, Tehran University of Medical Sciences, Tehran, Iran; 4 Department of PediatricNephrology, Bahrami Children Hospital, Tehran University of MedicalSciences, Tehran, Iran

**Correction to: Lipids Health Dis 19, 65 (2020)**

**https://doi.org/10.1186/s12944-020-01245-3**

Following publication of the original article [1], the authors noticed a mistake in the surname of Dr. Sayed Yousef Mojtahedi. In the published version, it is Motahedi and should be corrected to Mojtahedi. The original article has been updated.
